# Effects of Online Problem-Solving Instruction and Identification Attitude Toward Instructional Strategies on Students' Creativity

**DOI:** 10.3389/fpsyg.2021.771128

**Published:** 2021-10-14

**Authors:** Yi-Ping Wang

**Affiliations:** College of International Relations, Huaqiao University, Xiamen, China

**Keywords:** online problem, instructional strategies, identification attitude, affective component, creativity

## Abstract

Problem-solving ability is an essential part of daily life. Thus, curiosity and a thirst for knowledge should be cultivated in students to help them develop problem solving and independent thinking skills. Along with positive attitudes and an active disposition, these abilities are needed to solve problems throughout the lifespan and develop -confidence. To achieve educational objectives in the context of globalization, creative ability is necessary for generating competitive advantages. Therefore, creative thinking, critical thinking, and problem-solving ability are important basic competencies needed for future world citizens. Creativity should also be integrated into subject teaching to cultivate students' lifelong learning and a creative attitude toward life. A questionnaire was distributed to 420 students in colleges and universities in Fujian, China. After removing invalid and incomplete responses, 363 copies were found to be valid yielding a response rate of 86%. Findings indicate that the new generation requires high levels of support to develop creativity and integrate diverse subjects such as nature, humanities, and technology. A rich imagination is needed to root creativity in the new generation.

## Introduction

Problem solving is ubiquitous in modern life and an essential skill for overcoming the problems we encounter daily. Problems can be overcome using problem-solving principles and creative inspiration from individuals (Hao et al., 2016). Thus, students' curiosity and thirst for knowledge should be cultivated to develop their problem solving and independent thinking abilities. An active approach and positive attitude to solving problems may enhance self-confidence and the ability to cope with challenges.

Education aims to cultivate healthy personalities, thinking, judgment, and creativity (Su et al., 2014). Essentially, education is the learning process to expand students' potential and cultivate their ability to adapt to—and improve—their environment. Basic goals of education should include self-expression, independent thinking, active inquiry, and problem solving. The curriculum goals should be life-centered to develop individuals' potential, cultivate scientific knowledge and skills, and help students adapt to the demands of modern life (Atmatzidou et al., 2018). Education aims to deliver basic knowledge, cultivate physical and mental development, inquiry, and reflection, and create healthy citizens through activities involving interaction between individuals, individuals and society, and society and nature. To achieve educational objectives, students should be guided to develop their performance and creation abilities, research and active exploration abilities, independent thinking and problem-solving abilities. In the current globalization context, creative abilities are required for building competitive advantages. Accordingly, creative thinking, critical thinking, and problem-solving abilities are key skills for future world citizens. The cultivation of creativity should also be integrated into subject instruction, so that students develop their lifelong learning and creative attitudes toward life. Many countries are eager to cultivate creative new generations and promote the development of local business and humanistic technological education. It has become a national platform for the new generations of international technological art (Zhang and Chu, 2016). In particular, the traditional productivity-oriented competition model is slowly being transformed to creativity-oriented industries. Innovation capability is likely to bring competitive advantages in the Internet information age. As the field of information technology grows exponentially, innovation capability has become more important. However, if opportunities for development are missed, it can be difficult to catch up as the need for creativity is likely to grow in the foreseeable future.

In this study we focus on student creativity and how it is affected by online problem-solving instruction and identification of attitudes toward instructional strategies. Our purpose is to help the new generation develop creativity and a rich imagination to integrate the power of nature, humanities, and technology.

## Literature Review and Hypothesis

Su et al. (2017) proposed that teachers who use effective instructional strategies allow students to successfully negotiate the challenges of life, as effective instructional strategies may enhance students' problem-solving ability. Deeper relationships between teachers and students also result in better learning motivation for students. Art-related activities were used to observe the factors affecting preschool children's problem-solving ability (Calvo et al., 2018). These factors included the cognition of problem goals, the development of perception ability, individual experience, interaction among peers, and resource assistance provided by teachers' instructional strategies. (LaForce et al., 2017) pointed out that identifying problem-related data is an essential step in the problem-solving process, i.e., the process of acquiring data, judging data, reducing data coverage, or linking relevant data (Wu et al., 2020a). Teachers' instructional strategies for online problem solving also affect student performance. The following hypothesis was therefore established for this study.

H1: Online problem-solving instruction has a significant positive correlation with identification attitude.

Lu et al. (2017) consider that teachers can enhance students' problem-solving ability and cultivate their problem-finding skills through instructional strategies guiding discussion of current affairs. Instructional strategies and the use of multimedia in technology education can induce students' identification attitudes and learning motivation, ultimately enhancing learning effectiveness and facilitating the development of imagination and creativity. Students with identification attitudes toward strategies could design problem-solving methods using science (Newhouse, 2017). The students understood that innovation was not necessarily the novel creation of “something from nothing” but might involve modification and new development based on existing affairs (Wu et al., 2020b). Achilleos et al. (2019) regard attitude toward education instructional strategies as the most important factor in students' creativity learning, where teachers, social and cultural factors, and experience in learning a foreign language revealed significant correlations. Our second hypothesis was therefore presented for this study.

H2: Identification attitude shows strong positive correlations with creativity.

Hsieh et al. (2017) posit that science-related thinking, discovery, and creation can be regarded as the research component of problem solving. Creativity is characterized by keenness, fluency, flexibility, originality, and elaboration—a kind of mental intelligence to generate distinct new concepts from known experiences or knowledge to solve problems with creative methods. Creativity can also be the application of known information, based on targeted outcomes, to generate novel, unique, and valuable new concepts or a new product or technology, unexplored innovative concepts or problem-solving abilities (Wu et al., 2021). Joachim et al. (2018) consider creativity as a part of problem solving, as problem-solving characteristics often involve novel thinking, strong motivation and determination to present the important status of the solution in the latent process of problem solving. However, Joachim et al.'s (2018) views on creativity and problem-solving have largely been unexplored to date. Novel performance at any level of the creative process could be considered as creation. Rietz et al. (2019) stated that life brings diverse problems and the key to addressing these lies in creativity. Only when people invest more attention in creativity can problems be solved leading to optimum solutions for life's challenges. This gives rise to our third hypothesis.

H3: Online problem-solving instruction reveals strong positive correlations with creativity

## Methodology

### Operational Definitions

#### Online Problem-Solving Instruction

Referring to Chen et al. (2019), the dimensions of online problem-solving instruction in this study were as follows.

1. Exercise example: Examples to illustrate teaching goals are provided as part of teachers' instruction. Students can learn effective problem-solving skills by observing experts' problem-solving interpretation and demonstration step-by-step.

2. Problem orientation: Problem-oriented learning refers to teachers giving carefully-designed situational problems to students, who start from a problem and proceed to problem solving and learning. After self-learning, students participate in team discussion or discussions with teachers. With constant trials, solutions are eventually proposed.

#### Identification Attitude

The dimensions for identification attitude toward learning are based on Tang et al. (2019) and contain the following three components.

1. Cognitive component: This refers to an individual's belief in or knowledge of specific matters. The cognition of attitude refers to evaluation of meaning from factual statements presented, i.e., an individual may form an attitude for or against a particular object. For instance, students understand that teachers have rich professional knowledge and can present materials with good organization.

2. Affective component: The affective or emotional component refers to an individual's emotions and feelings, including positive and negative feelings of respect and contempt, like and dislike, sympathy and exclusion. For example, students evaluating a teacher as a friendly person would have positive feelings about the teacher and want to develop that relationship.

3. Behavioral component: Behavior refers to an individual's response tendency to attitude objects, i.e., an individual's explicit behavioral performance when acting in relation to objects. Possible responses include approach, avoidance, or indifference. For instance, students might accept their teachers' arrangement of an activity with respect and actively ask teachers questions.

#### Creativity

Kim et al. (2019) consider creativity includes basic cognitive abilities of divergent thinking, and that such abilities can be understood through testing tools or observation.

1. Fluency: Fluency refers to the quantity of a person's concept output, i.e., the ability to generate possible programs or solutions. A student with fluent thinking would propose several responses at the concept generation stage.

2. Flexibility: Flexibility is the ability to change thinking direction, i.e., being able to think of different methods when problems occur, to find out distinct applications or new concepts.

3. Originality: Originality refers to generation of unique and novel ideas, i.e., doing unexpected things or having the ability to see others' points of view.

4. Elaboration: Elaboration is a supplementary idea that refers to the ability to add new ideas to an original concept, i.e., the ability to increase novel concepts or build on existing ideas or basic concepts.

### Research Objective

There are 89 colleges and universities in Fujian, China (50 colleges and 39 universities). Students in these institutions in Fujian comprised the research sample, and we distributed 420 copies of our questionnaire to them. After removing invalid and incomplete questionnaires, a total of 363 valid copies were returned, with a response rate of 86%.

### Analysis

This research focused on discussing online problems about teaching and teaching strategies. It used experimental design and online problem solving to do experimental research for 2 hours every week for 24 weeks (48 hours in total). To analyze data from the questionnaire, Structural Equation Modeling (SEM) was used. We followed a two-stage analysis of goodness-of-fit and model verification. Confirmatory Factor Analysis (CFA) was first executed, aiming to test complex variables in the model by deleting measured variables with negative effects on the cause-and-effect analysis. We then proceeded with path analysis with the modified model. Path analysis aims to estimate the path relationship among variables. Without testing complex variables through CFA, the path analysis might be affected by complex variables resulting in poor goodness-of-fit or an insignificant model path. Amos 18.0 was used in this study for the model fit test. The measurement result of CMIN/DF is considered good if lower than five and excellent if lower than three; Goodness-of-Fit Index (GFI), Adjusted Goodness-of-Fit index (AGFI), Normed Fit Index (NFI), Incremental Fit Index (IFI), Tucker-Lewis Index (TLI), and Comparative Fit Index (CFI) are considered good if higher than 0.9; and Root Mean Square Residual (RMR), Root Mean Square Error of Approximation (RMSEA), and Standardized Root Mean Square Residual (SRMR) are good if values are lower than 0.05.

## Results

### Factor Analysis

Two factors of “exercise example” (eigenvalue = 4.638, α = 0.88) and “problem orientation” (eigenvalue = 3.751, α = 0.85) were extracted from the scale of instructional strategies for online problem solving. The cumulative covariance accounted for was 72.683%. Three factors were extracted from the identification attitude scale: “cognitive component” (eigenvalue = 2.463, α = 0.81), “affective component” (eigenvalue = 1.754, α = 0.83), and “behavioral component” (eigenvalue = 1.491, α = 0.84). The cumulative covariance reached 73.942%. Four factors were extracted from the creativity scale: “fluency” (eigenvalue = 2.461, α = 0.84), “flexibility” (eigenvalue = 2.055, α = 0.82), “originality” (eigenvalue = 1.976, α = 0.87), and “elaboration” (eigenvalue = 1.689, α = 0.86). The cumulative covariance accounted for was 79.317%.

### Empirical Analysis of SEM

CFA results indicated the convergent and discriminant validity of the model were first observed, with convergent validity describing the reliability of individually observed variables, construct reliability (CR), and average variances extracted (AVE). Values of more than 0.5 indicate good reliability of individually observed variables. The factor loadings of the observed variables in the empirical analysis model were higher than the suggested value. CR should exceed 0.6, although some researchers suggest that 0.5 or above is acceptable. The model calibration results reveal CR was higher than 0.6, and AVE higher than 0.5, thus conforming to the suggested values.

**Figure 1:**
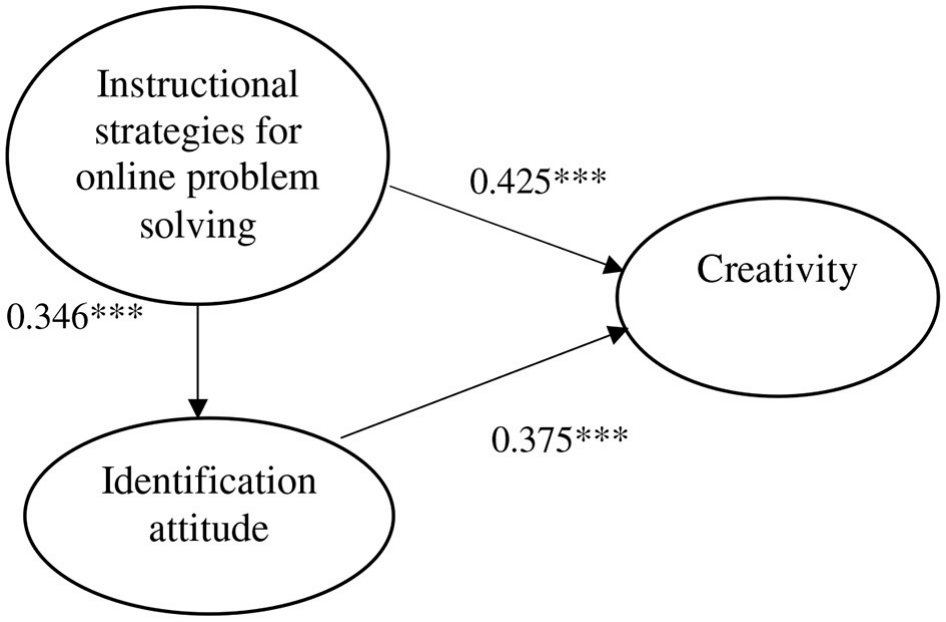
FigureModel path diagram.

Regarding the calibration results of structural equations, χ^2^^/^^*df*^, RMSEA, GFI, AGFI, RMR, and NFI were also calculated. For χ^2^^/^^*df*^ a standard ≦5 is suggested and χ^2^^/^^*df*^ = 2.422 ≦ 5 in this study. The standard for RMSEA is ≦0.08; reported here as 0.044 ≦ 0.08. GFI has a suggested standard of ≧0.9 and here it is reported as 0.951 ≧ 0.9. AGFI's suggested standard ≧0.9; it shows AGFI = 0.927 ≧ 0.9 in this study. RMR has a suggested standard of ≦0.05, and here it was reported as 0.023 ≦ 0.05. The NFI standard is ≧0.9; here it presents NFI = 0.937 ≧ 0.9 in this study. The overall model fit is good. The parameter calibration of the structural equation is shown in Table 1 and Figure 1. The research results reveal instructional strategies for online problem solving → identification attitude: 0.346^***^, that is, H1 was supported. Identification attitude → creativity: 0.375^***^, that is, H2 was supported, and instructional strategies for online problem solving → creativity: 0.425^***^, that is, H3 was supported.

**Table 1:** Structural equation modeling result.

**Parameter/evaluation standard**	**Coefficient**
Instructional strategies for online problem solving → identification attitude	0.346^***^
Identification attitude → creativity	0.375^***^
Instructional strategies for online problem solving → creativity	0.425^***^
χ2/degree of freedom ≦5	2.422
Root mean square error of approximation (RMSEA) ≦0.08	0.044
Goodness-of-fit index (GFI) ≧0.9	0.951
Adjusted goodness-of-fit index (AGFI) ≧0.9	0.927
Root mean square residual (RMR) ≦0.05	0.023
Normed fit index (NFI) ≧0.9	0.937

*** *p < 0.001*.

## Discussion

The results show that online instructional strategies for online problem solving can enhance students' creativity. Apparently, an expository teaching style is no longer sufficient to cope with challenges encountered. Rather, teachers need to be willing to constantly learn and change their teaching behavior to cope with the rapid development of new technology and enhance teaching efficiency. When conveying new knowledge to beginners, the provision of exercise examples may help students establish new schema to benefit the application to similar situations. When lacking relevant schema, beginners may try to solve problems with trial and error. In this case, exercise examples with experts demonstrating problem-solving steps could benefit students' learning performance in the new field. Problems studied in real life may facilitate students' creativity, drawing on their existing knowledge as they use available resources and unconsciously apply existing knowledge to enhance creative ability. The solutions to problems are unpredictable but require the ability to cope with interaction between people in a given culture or society in different situations. As a result, teachers should make decisions with the consideration of situational changes in the teaching site, i.e., students' ability, performance and teaching schedule, rather than generalizing across all situations. We do not suggest limiting creative thinking or defining set times for enhancing students' creative thinking. Instead, factors that influence creative efficiency, creative value, and curriculum schedules should be taken into account. As teachers plan their teaching activities, they should pay particular attention to students' academic performance and the vicarious experience of teachers or peers. Uysal (2014) believed people can develop their mental ability through learning even without any creative invention. When we face any new concept, it is better to keep an open mind. That way we will realize there is still a lot to be created (Fernández et al., 2018). Labusch et al. (2019) said the development of creativity is not only creating positive thoughts but also turning these advantages into something more refined and broader. Teachers need to provide learning opportunities that students can apply in their daily lives leading to a re-evaluation of their identification attitudes toward instructional strategies. In this case, enhancing students' self-efficacy may assist them in overcoming learning challenges and cultivating a more positive learning attitude.

## Conclusion

The research results demonstrate that online problem solving supports students to examine their ideas, chase after knowledge and continually improve their learning. They can freely develop their imaginations and make choices without being limited to find tools suitable for self-performance. They can concentrate on details, retain memories, and calmly think of more elaborate problem-solving approaches. Students draw on plans and organization to make significant progress in their thinking depth, novelty, flexibility, unique style, and diversity of function. To cultivate students' habits of brainstorming and thinking, they must become familiar with the general use of contextual information, and flexibility to change approaches and seek answers. Training flexible thinking is essential so that students can cope with problems with ease, propose various options and generate solutions. Lumsdaine and Lumsdaine (1995) let students learn from each other and modify their own thought. This transition could help them to achieve their potential. Solitary and monotonous learning material can no longer attract students' attention. Teachers need to provide a wider variety of materials and free choices without limit. They could also find more suitable tools for teaching. Therefore, Treffinger and Isaksen (1992) no longer provide model answers. They want students to explore and develop without any restriction. This could also amplify their personal experience and bring more options into it. It enhances student's uniqueness, and this needs overall growth, subjectively and objectively. People should never venerate one over the other. We should also learn to make good use of the conditions and things we already have. The same thing could have an entirely different outcome depending on how we use it (Aşik and Erktin, 2019). Consequently, problem-solving instruction could assist in the cultivation of creativity in students' practice ability or cultivation of independent thinking and problem-solving ability. Teachers should attempt to create beneficial educational environments, cultivating students' learning interests, and enhancing their mental development. With accumulated experience, students can then be encouraged to develop more flexible skills, sensitive perception, and active thinking along with the ability to appropriately express these experiences. This would provide comprehensive preparation for enhancing students' creative thinking ability. Instructional strategies for online problem solving heavily emphasize cooperative discussion, brainstorming, and presentation. Tasks focusing on students' favorite novels and other relevant interests are valuable for sustaining long-term attention. Success in learning does not simply rely on rich knowledge and skillful techniques; affective attitudes also play an important part. Such characteristics may encourage students to positively and actively face problems and logically enhance their learning attitudes step-by-step.

## Data Availability Statement

The raw data supporting the conclusions of this article will be made available by the authors, without undue reservation.

## Ethics Statement

The studies involving human participants were reviewed and approved by the Ethical Committee of the Huaqiao University. The patients/participants provided their written informed consent to participate in this study.

## Author Contributions

Y-PW performed the initial analyses and approved the submitted version of the manuscript.

## Conflict of Interest

The author declares that the research was conducted in the absence of any commercial or financial relationships that could be construed as a potential conflict of interest.

## Publisher's Note

All claims expressed in this article are solely those of the authors and do not necessarily represent those of their affiliated organizations, or those of the publisher, the editors and the reviewers. Any product that may be evaluated in this article, or claim that may be made by its manufacturer, is not guaranteed or endorsed by the publisher.
